# Metabolic signatures associated with oncolytic myxoma viral infections

**DOI:** 10.1038/s41598-022-15562-3

**Published:** 2022-07-23

**Authors:** Rohit Mahar, Mukundan Ragavan, Mario C. Chang, Savannah Hardiman, Nissin Moussatche, Adam Behar, Rolf Renne, Matthew E. Merritt

**Affiliations:** 1 Department of Biochemistry and Molecular Biology, University of Florida College of Medicine, Gainesville, FL USA; 2 Department of Molecular Genetics and Microbiology, University of Florida College of Medicine, Gainesville, FL USA

**Keywords:** Metabolomics, Cancer metabolism

## Abstract

Oncolytic viral therapy is a recent advance in cancer treatment, demonstrating promise as a primary treatment option. To date, the secondary metabolic effects of viral infection in cancer cells has not been extensively studied. In this work, we have analyzed early-stage metabolic changes in cancer cells associated with oncolytic myxoma virus infection. Using GC–MS based metabolomics, we characterized the myxoma virus infection induced metabolic changes in three cancer cell lines—small cell (H446) and non-small cell (A549) lung cancers, and glioblastoma (SFxL). We show that even at an early stage (6 and 12 h) myxoma infection causes profound changes in cancer cell metabolism spanning several important pathways such as the citric acid cycle, fatty acid metabolism, and amino acid metabolism. In general, the metabolic effects of viral infection across cell lines are not conserved. However, we have identified several candidate metabolites that can potentially serve as biomarkers for monitoring oncolytic viral action in general.

## Introduction

Cancer prevalence has increased mortality worldwide and in the US, accounting for almost 600,000 deaths each year in 2019 and 2020^1^. Current treatment paradigms involve combinations of chemotherapy, radiotherapy, and surgery, depending on the specifics of each case. However, treatment options are often limited depending on the stage of cancer development leading to a poor prognosis^2^. Recent advances in cancer treatment include stem cell therapies^3,4^ and immunotherapies^5–7^, including oncolytic viral therapy^8^.

Oncolytic viral therapy (or virotherapy) employs viruses to target and kill tumor cells while also triggering an immune response. Oncolytic viruses (OVs) from ten different families belonging to both DNA and RNA viruses have been utilized in oncolytic virotherapy^9,10^. Virotherapy offers a significant advantage as a therapeutic platform since it enables targeting tumor types that are resistant to chemotherapeutic or other immunotherapeutic approaches^11^. Furthermore, OVs either exhibit a natural response to attack cancer cells or can be genetically altered to confer specificity to attack tumor cells making this approach very attractive for treating different cancer types and at various stages^9^.

In addition to targeting the viruses directly, several studies have shown that the oncolytic viruses can be used in conjunction with other therapeutic agents to promote tumor cell death^12^. In one study, herpes simplex virus (HSV) was used in combination with 5-fluorouracil to improve survival of animal models of gallbladder and colon cancer^13^. Similarly, oncolytic measles vaccine virus was used to promote complete cell death post-treatment with therapy-induced senescence agents such as gemcitabine and doxorubicin, thereby preventing regain of proliferation activity in different cancer cell lines^14^. Several other combinations involving oncolytic viruses and drugs targeting/triggering ER stress (unfolded protein response)^15^ and re-activation of apoptosis^16^ have been demonstrated.

An important aspect of developing pharmaceutical agents to target the cancer cells in tandem with OVs is to gain understanding of the changes caused by the viral infection in the cells that are targeted. Understanding the OV infection induced metabolic changes will likely discover biomarkers that can then be used to either use existing metabolic modulators and/or develop novel pharmaceutical agents to improve efficacy of treatment using OVs. Metabolomics, the systematic study of metabolites involved in various cellular processes, can readout changes in cancer metabolism and enables identification of potential biomarkers^17^ of treatment efficacy. Gas chromatography-mass spectrometry (GC–MS) is widely utilized in biomarker investigation, with ~ 50 years of established protocols for examining a wide range of metabolites including sugars, amino acids, organic acids, and fatty acids^18^. The unique mass spectral patterns of the molecules recorded with standardized parameters have been curated in mass libraries such as US National Institute of Standards and Technology (NIST)^19^, which makes GC–MS a universal tool for metabolomics. The extraordinary sensitivity and high-resolution afforded by GC–MS makes it a robust method to identify the metabolites involved in the perturbation of cancer cell homeostasis due to infection and/or treatment.

In this study, we have utilized GC–MS based metabolomics to characterize metabolic changes associated with myxoma virus infection in three cancer cell lines: lung cancer cells A549 (non-small cell) and H446 (small cell), and glioblastoma cells (SFxL). The OVs take advantage of cellular mechanism commonly found in cancer cells allowing them to infect and replicate within cancer cells while sparing normal or healthy cells. Therefore, we did not utilize normal cells in this study. Lung cancer causes more deaths in the US per capita than any other cancer. Glioblastoma has a mortality rate that exceeds almost any other cancer, with a survival expectancy of ~ 5% over 5 years. As such, the cell lines chosen represent some of the most dangerous cancers that are arguably the most important targets for alternative treatments. Multivariate statistical analysis was employed on metabolite data sets to identify the key metabolites that exhibit differences between control and myxoma infected cancer cells. To the best of our knowledge, this is the first study to examine the global metabolic changes in A549, H446, and SFxL cells caused by oncolytic myxoma virus.

## Results

### GC–MS analysis of the H446, A549, and SFxL cancer cell lines

**Figure 1:**
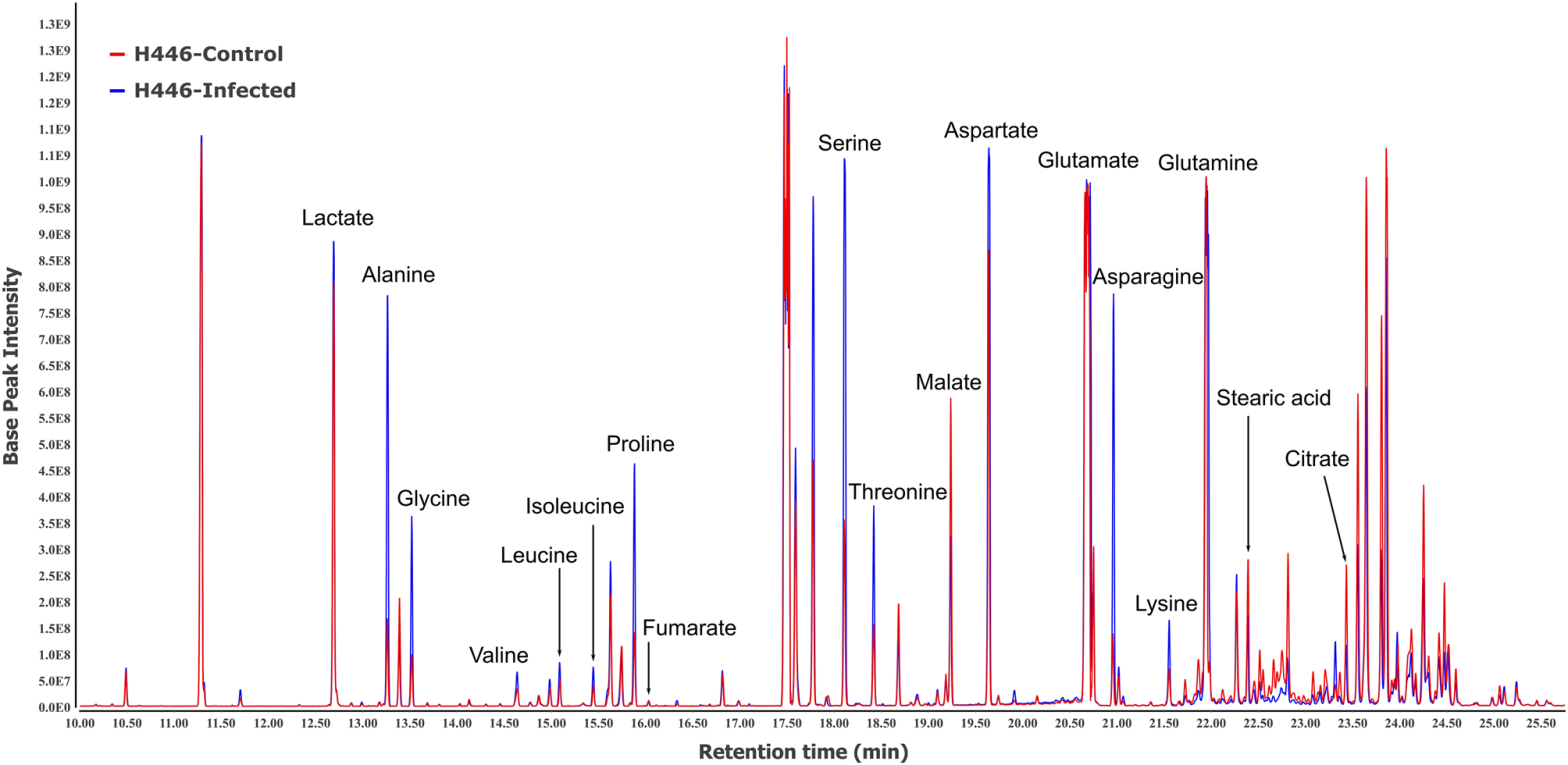
Representative chromatograms of H446 cancer cells: GC–MS profile of H446 control (red) and infected (blue) cells. The chromatographic peaks of the metabolites are labeled in the overlaid chromatograms of H446 control and infected cells. Qualitatively, levels of lactate, alanine, proline, serine, aspartate, asparagine, leucine, and isoleucine are different between control and infected groups.

**Figure 2:**
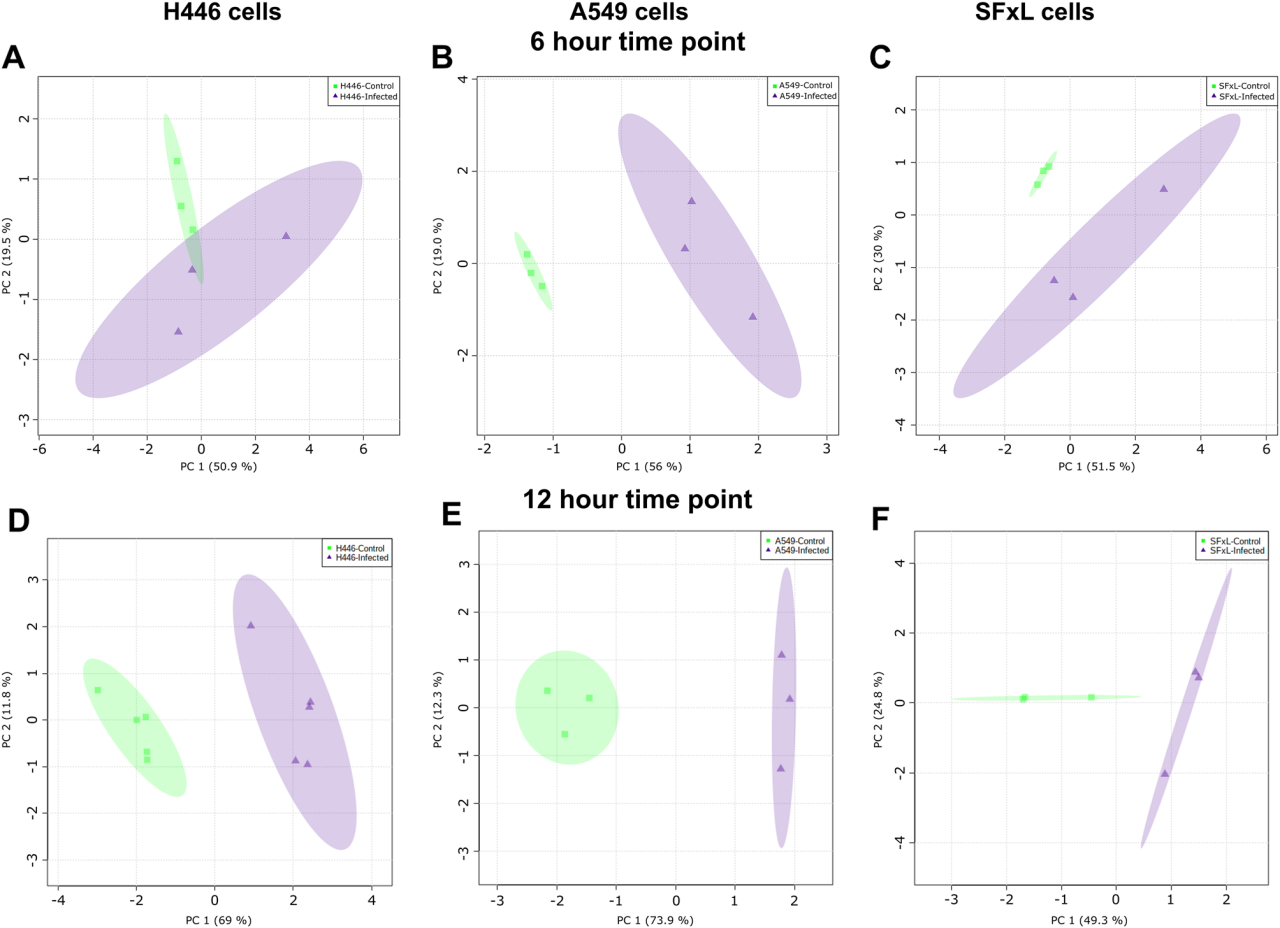
Unsupervised multivariate analysis of control and infected cells: 2D scores plots of the principal component analysis (PCA) from GC-MS data for H446, A549, and SFxL cancer cells at 6 h post infection panels (**A**), (**B**), and (**C**), respectively and 12 h post infection panels (**D**), (**E**), and (**F**), respectively. The amount of variance explained for PC 1 and 2 are displayed in parentheses. The shaded areas indicate the confidence regions (95%) based on the data points for each condition in the PCA models. The unsupervised PCA models suggest a powerful effect due to myxoma infection.

The GC–MS profile of H446 control and infected cells demonstrate the metabolic effects of OV action (Fig. 1). A visual comparison readily shows differences in metabolites corresponding to different pathways with several of them present in elevated levels in the infected cells. PCA was performed to examine the differences between control and infected H446, A549, and SFxL cancer cells at 6 and 12 h post infection (Fig. 2). The two-dimensional (2D) scores plots in Fig. 2A and D demonstrate the clear separation between H446 control and infected cells at 6 and 12 h. PC1 and PC2 showed variances of 50.9% and 19.5% as well as 69% and 11.8%, respectively. Similarly, 2D scores plots in Fig. 2B,C,E, and F show excellent separation between control and infected groups of A549 and SFxL cells at both time points.

**Figure 3:**
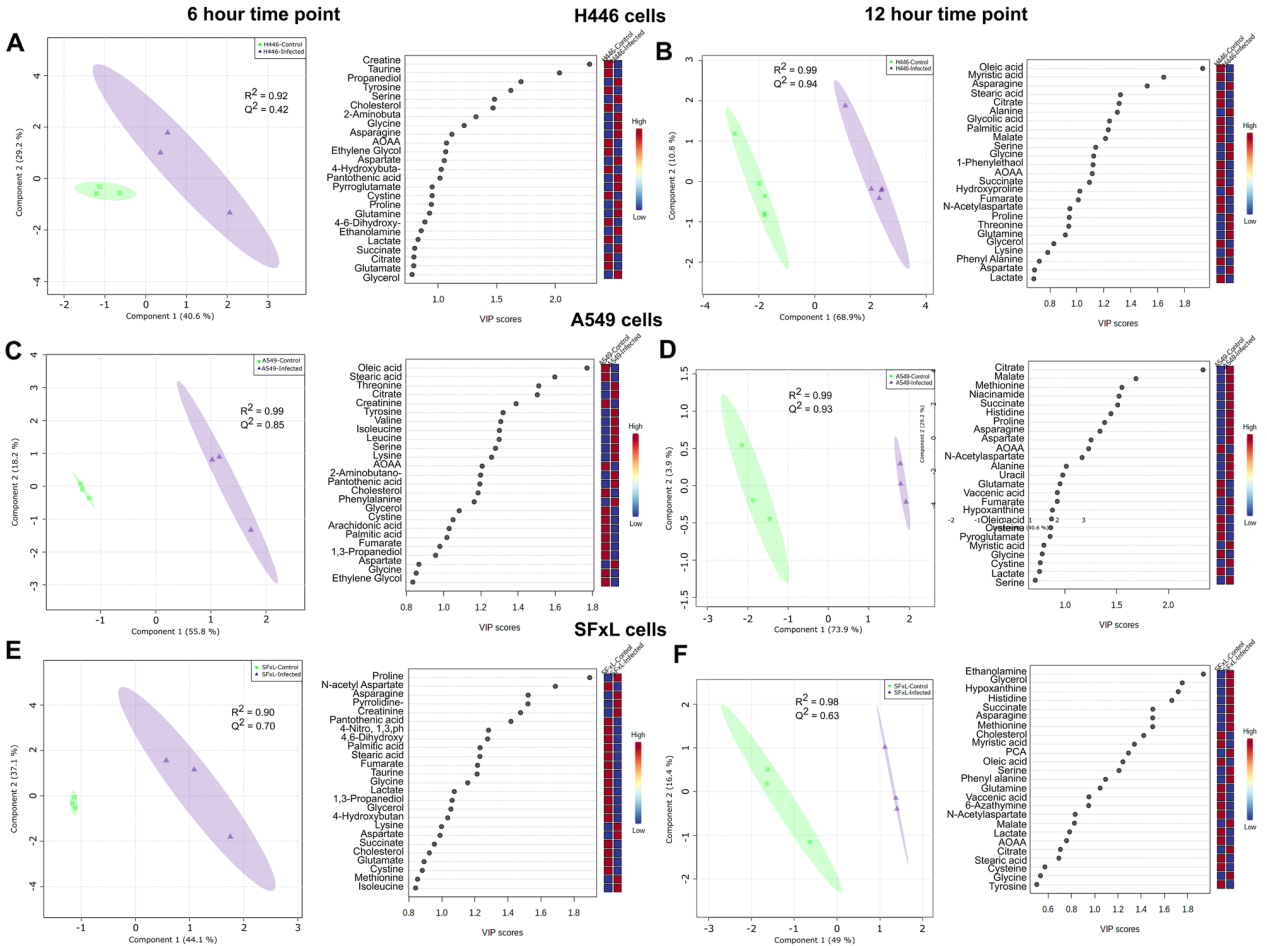
Supervised PLS-DA of control and infected cells: 2D scores plots of PLS-DA and VIP plots for three cancer cell lines at 6 h and 12 h post infection—(**A**,**B**) H446 cells, (**C**,**D**) A549 cells and (**E**,**F**) SFxL cells. The explained variance for components 1 and 2 are displayed in parentheses on each axis. The shaded areas reflect the 95% confidence regions based on the data points for each group in PLS-DA models. VIP scores plots from the PLS-DA demonstrating the differences in the level of top 25 cellular metabolites between control and infected cancer cells. Abbreviations: AOAA: (Aminooxy)acetic acid, P.C.A.: pyrrolidine 1,2-carboxylic acid.

To strengthen the separation between control and infected cancer cells and validate the PCA, a supervised PLS-DA classification method was utilized. 2D scores plots from PLS-DA analyses at 6 h post infection are shown in Fig. 3A,C, and E for H446, A549, and SFxL cells. For all three cell lines (Fig. 3A,C, and E), R^2^ and Q^2^ were similar while values for R^2^ was close to unity suggesting both robustness and good predictive accuracy with 2 components of the PLS-DA model. Even greater separation and robustness was observed in the 2D scores plot of all cell lines at 12 h post infection.

The VIP scores for the top 25 metabolites obtained from PLS-DA analyses of H446, A549, and SFxL cells indicated robust changes in metabolic profiles at 6 h and 12 h post infection (Fig. 3). At the 6 h time point, SFxL and A549 cancer cells showed excellent separation between control and infected groups in both PCA and PLS-DA scores plot. However, H446 cells displayed less separation between control and infected groups (Fig. 3A). In H446 cells, 40% of the top 25 metabolites were common at both time points with similar increases and decreases in metabolite levels (Fig. 3A and B). At the 12 h time point, H446 cells contained several fatty acids (only stearic acid and palmitic acid were present in the cell culture media) and citric acid cycle intermediates that were higher in control while various amino acids were found elevated in infected cells (Fig. 3B). In A549 cells, 32% of the top metabolites were found in both 6 and 12 h time points (Fig. 3C and D). Interestingly, metabolites associated with energy metabolism were higher in infected A549 cells while metabolites predominantly associated with amino acid metabolism were higher in control cells (Fig. 3D). The corresponding VIP scores plot of a PLS-DA model of SFxL cells, depicted the higher levels of ethanolamine, glycerol, hypoxanthine, succinate, and few amino acids such as histidine, methionine, and asparagine in SFxL MYXV infected cells. Cholesterol, myristic acid, oleic acid, vaccenic acid, glutamine, lactate, and (Aminooxy) acetic acid (AOAA) were found at higher levels in SFxL control cells, some of which accounted for 36% of VIP scoring metabolites (Fig. 3E and F). Changes in metabolite levels due to oncolytic myxoma infection of all three cell lines as identified by VIP scores are shown quantitatively in Figures S1–S3.

### Classification between control and infected cancer cells

**Figure 4:**
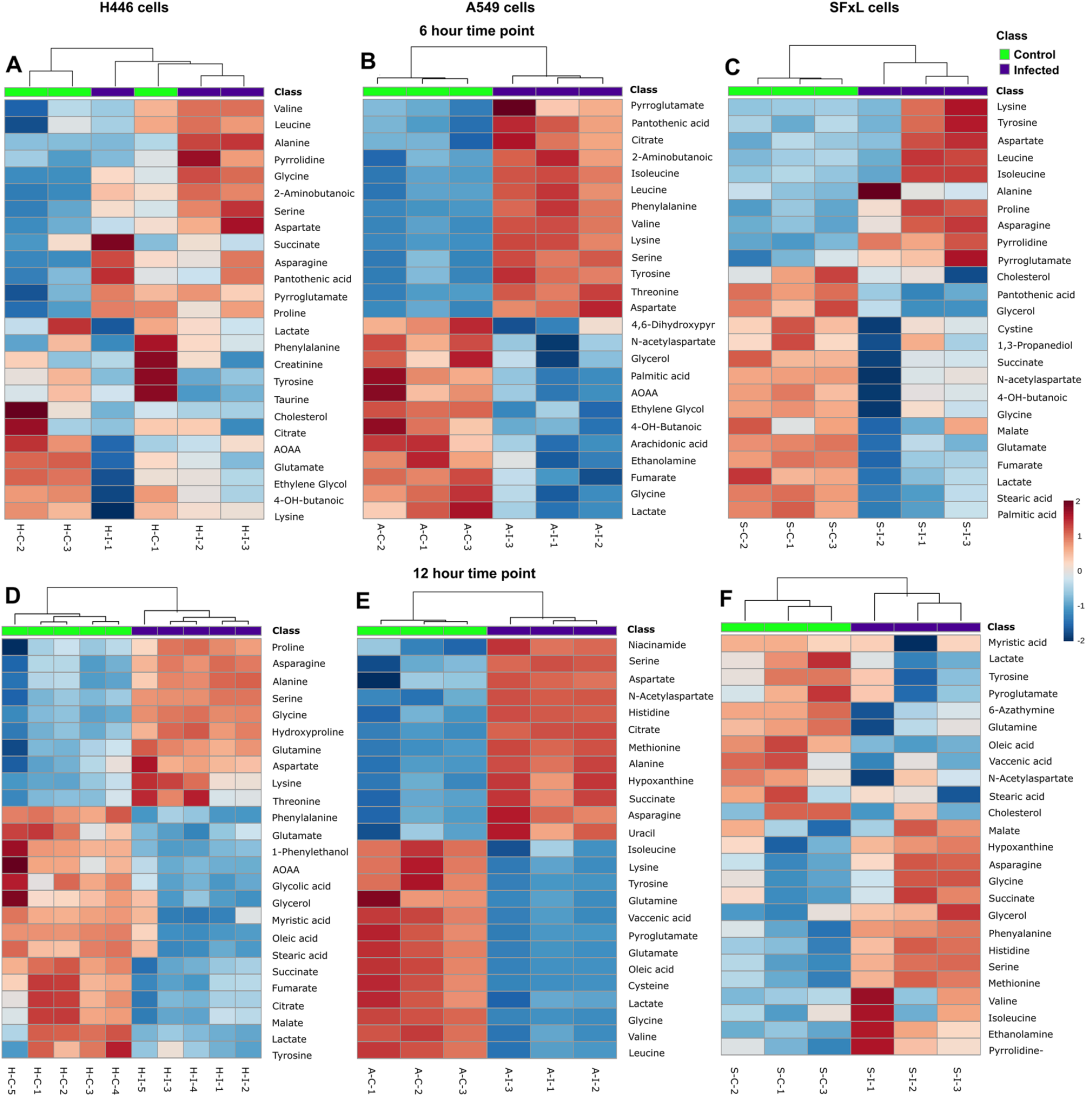
Demonstration of clustering between control and infected cells: Hierarchical clustering results are shown as heatmaps demonstrating the differences in the levels of top 25 metabolites (identified via Student’s t-test in Metaboanalyst) between control and infected H446, A549, and SFxL cells at 6 h (**A**,**B**, and **C**, respectively) and 12 h (**D**,**E**, and **F**, respectively).

Results from clustering analysis of the heatmaps using the top 25 metabolites identified using t-tests in H446 cells shows excellent classification of individual samples into control and infected groups at 6 and 12 h time points (Fig. 4A and D). Similar results were obtained for A549 cells (Fig. 4B and E) and SFxL cells (Fig. 4C and F) as well, although the SFxL cells showed higher biological variability at the 12 h time point.

### Metabolite set enrichment analysis

**Figure 5:**
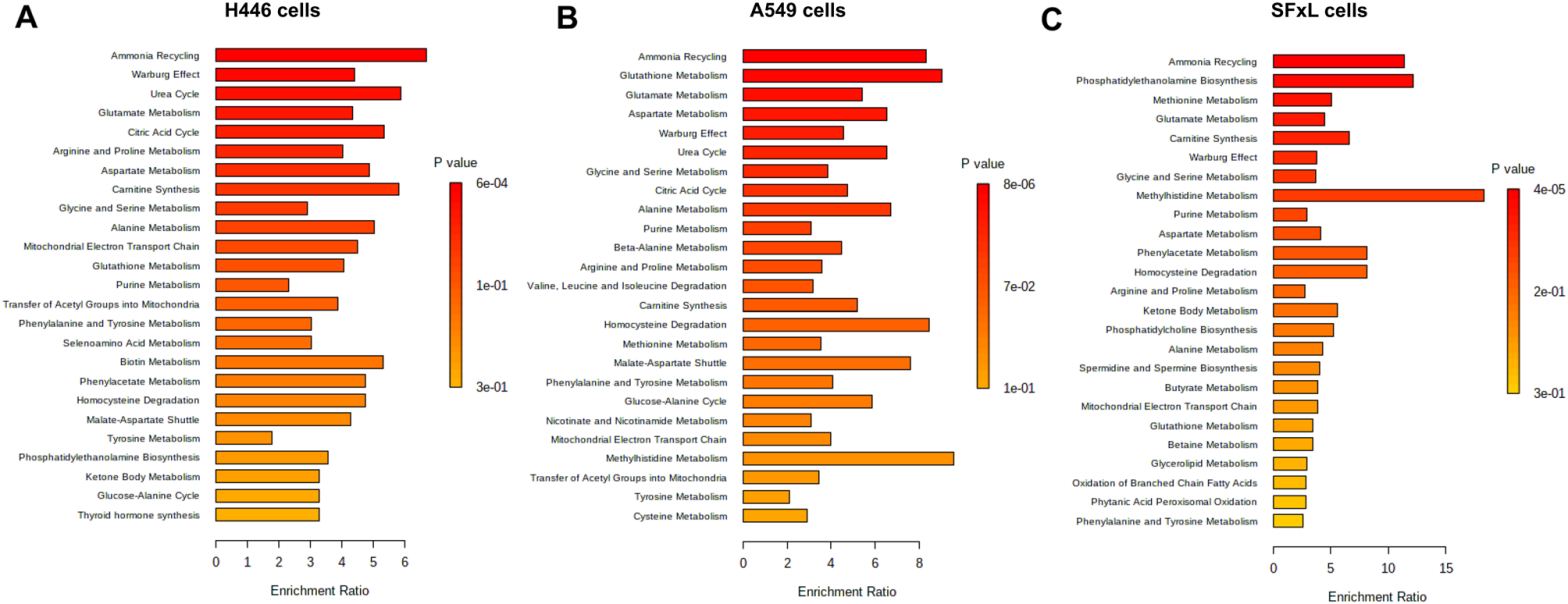
Investigation of enriched metabolic pathways: Metabolite Set Enrichment Analysis (MSEA), using significantly different metabolites between control and infected cancer cells. Overview of the top 25 enriched pathways for H446, A549, and SFxL cells in the panels, (**A**),(**B**), and (**C**), respectively. MSEA investigates if a group of functionally or metabolically related metabolites is significantly enriched. It also eliminates the requirement of to preselect metabolites based on some cut-off threshold.

Statistically different metabolites (identified via Student’s T-test) between control and infected cells were used for metabolite set enrichment analysis (MSEA). The MSEA results demonstrated an overview of perturbed pathways due to MYXV infection in H446, A549, and SFxL cancer cell lines (Fig. 5). Based on the enrichment ratio and p-values shown as a bar graph for H446 cells, the pathways related to ammonia recycling, Warburg effect, urea cycle, citric acid cycle, and amino acid metabolism such as glutamate, arginine, proline, and aspartate were significantly enriched (Fig. 5A). Similarly, enrichment ratio and p- values shown for A549 cells suggested that the pathways of ammonia cycling, glutathione, glutamate, and aspartate metabolism were significantly enriched. Additionally, Warburg metabolism, urea cycle, glycine and serine metabolism, citric acid cycle and alanine metabolism were significantly enriched (Fig. 5B). The overview of the top 25 enriched pathways for SFxL cells showed that ammonia cycling and phosphatidylethanolamine biosynthesis pathways were enriched significantly with the enrichment ratio > 10 and methyl histidine metabolic pathway is significantly enriched with the enrichment ratio greater > 15 (Fig. 5C). The interactive pathway network displayed a global overview of enriched pathways along with metabolic networks for H446, A549, and SFxL cancer cell lines (Figures S4−S6).

### Common metabolites

**Figure 6:**
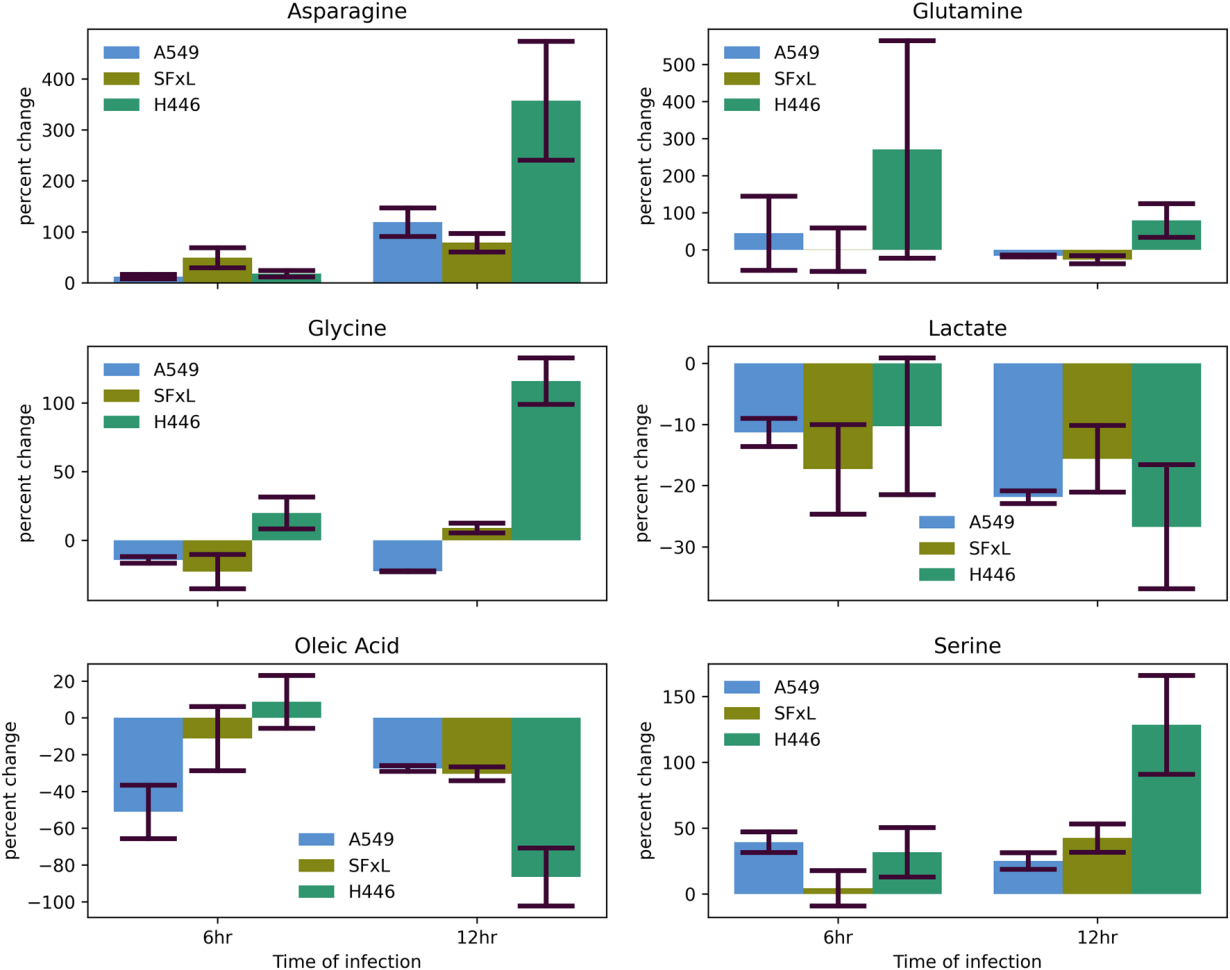
Metabolic signatures between control and infected cancer cells: Percent change in metabolite levels between control and MYXV-infected cells at 6 h and 12 h post infection. Percent change calculated as change from control to infected. Only those metabolites that were identified as statistically significant by Student’s t-test (p < 0.05) across all three cells (A549, SFxL, H446: N = 3) are shown here.

Among all the metabolites identified (Table S1) that were significantly different (Figure S1–S3), six metabolites were common across all three cell lines. Of these six metabolites (Fig. 6), asparagine, lactate, oleic acid, and serine manifested a similar behavior across all three cell lines and both time points. On the other hand, changes in levels of glutamine and glycine were not conserved in response to myxoma infection. Interestingly, lactate levels were lower in the infected group in all three cells. Similarly, oleic acid pool size was also decreased in response to MYXV infection, except in the case of H446 at the 6 h time point. In contrast, asparagine and serine levels were found to be increased in the MYXV infected cells. These changes suggest the effect of MYXV infection on a range of pathways including the whole of energy metabolism as well as amino acid and one-carbon metabolism.

### Infectivity

**Figure 7:**
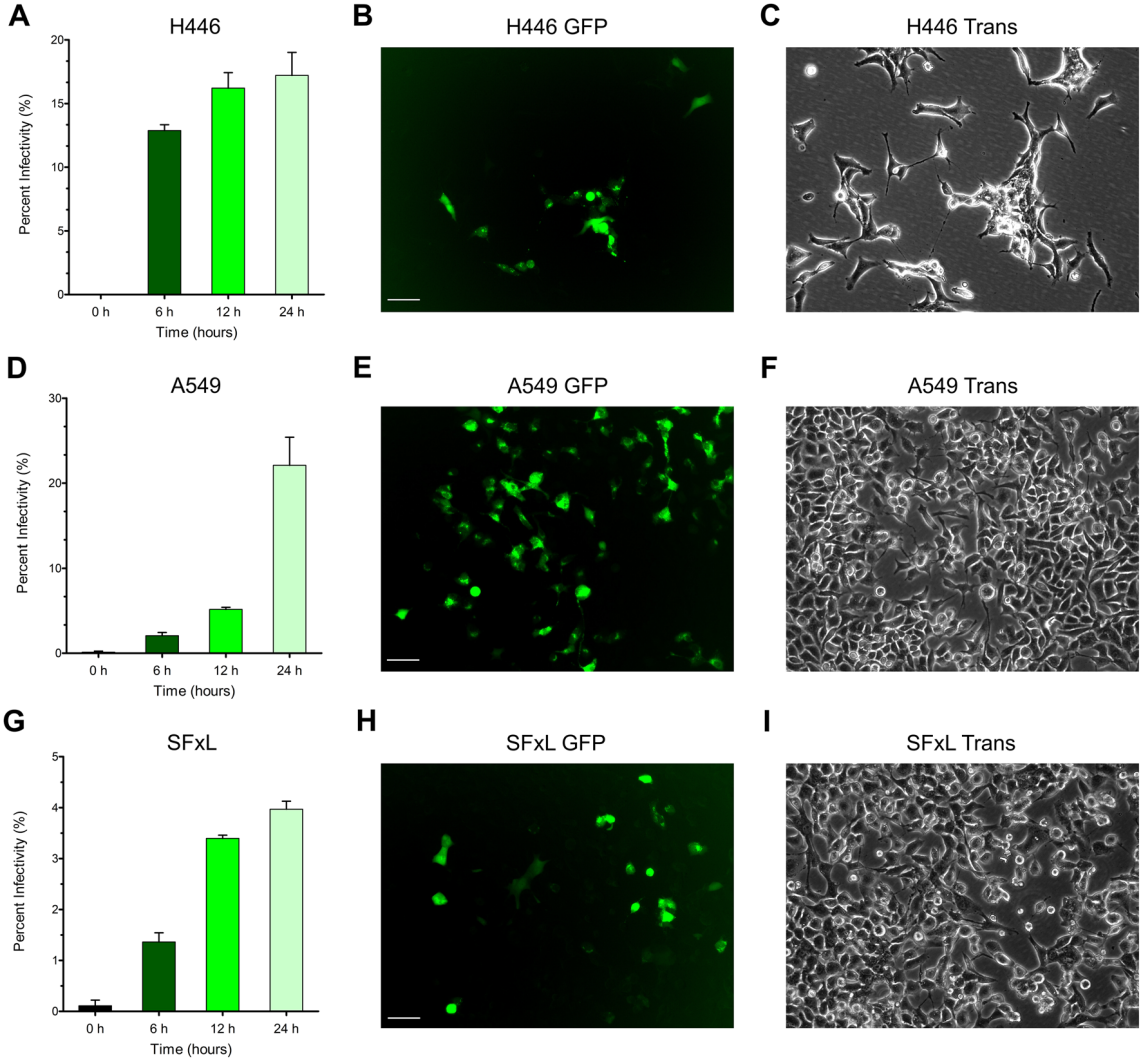
Time course assessment of myxoma virus infectivity: Percent infectivity reported over a 24 h period for all three cell lines (H446, A549, SFxL: N = 3). The percent of infected cells was determined by quantitating GFP signal captured with fluorescence microscopy. Representative images of FITC (GFP) and Transfluorescent (Trans) channels at 24 h are presented. The scale bar represents 50 μm.

The extent of infection was assessed by fluorescence microscopy imaging of GFP positive cells. MYXV was tagged with GFP to allow for the detection of infected cells. Across the span of 24 h all three cell lines showed a time dependent increase in the number of infected cells (Fig. 7). Calculated percent infectivity values showed that at 6 h H446 had the greatest percent of infectivity at ~ 13%, while A549 and SFxL had percents of ~ 2% and ~ 1.4%, respectively. At 12 h, H446 increased to ~ 16%, A549 to ~ 5.2%, and SFxL to ~ 3.4%. The percent infectivity values continued to increase through 24 h to values of ~ 17%, ~ 22%, and ~ 4% for H446, A549, and SFxL cells respectively. At 48 h, all living cells reached 100% infectivity, though this time point was characterized by extensive cell death.

### Extracellular metabolite analysis

Analysis of media samples showed decreases in the amount of glucose consumed in infected A549 and SFxL with no apparent difference in H446 cells (Figure S7). These glucose measurements align with extracellular metabolite analysis, which found significant decreases in the lactate excretion of infected A549 and SFxL cells (Figure S8). All infected cell lines showed increases in alanine secretion with significantly higher secretion observed in infected H446 and SFxL cells (Figure S8). Branched chain amino acids displayed significantly lower consumption in infected SFxL cells, while no significant differences in A549, and only a significant increase in the consumption of isoleucine in infected H446 cells (Figure S8). Extracellular aspartate levels were significantly higher in infected SFxL cells, trended higher in infected A549, and had no observable differences between control and infected H446 (Figure S8). There were so no significant differences in extracellular glutamine for all cell lines (Figure S8).

## Discussion

Myxoma virus (MYXV) has been studied for causing cancer cell death and cell proliferation inhibition in various cancer types^8^. Previously, it has been shown that the oncolytic activity of MYXV is due to viral induction of programmed cell death in some cancers, but none of the studies have targeted the metabolic changes due to MYXV infection in cancer cells^20–22^. In this study, we have investigated the initial metabolic changes due to MYXV infection in brain (SFxL), small cell lung cancer (H446), and non-small cell lung cancer (A549). While this panel could be expanded to include an even larger swath of cancers, this sampling covers some of the cancers that are otherwise most treatment resistant in all of oncology. Previous studies have shown that OVs take advantage of dysregulated pathways in cancer cells allowing them to infect and replicate there, while sparing normal cells^23–25^. This is an investigation of early stage metabolic changes in cancer cells (6 and 12 h post infection) due to myxoma infection. After 12 h post infection with MYXV, > 95% cells were alive in culture while cell survival 24 h post infection was less than 50% (data not shown) consistent with previous studies which also showed pronounced viral replication after 24 h^26,27^. More specifically, we assessed cell viability in all cell lines by cell counting and observed non-significant differences between control and infected cells at 6 h. At 12 h there were ~ 1.4 times more control A549 and SFxL cells compared to infected cells, and there were no significant differences observed between control and infected H446 cells. As the infection progressed, we observed a significant decrease in cell proliferation in all cell lines. Within the 12 h period, we observed a profound change in the metabolite profiles of MYXV infected (~ 3–16% infectivity with H446 having the greatest susceptibility to MYXV) cancer cells. We chose these time points as later time points begin to display larger amounts of cell death, which we judged to have negative impact on the reproducibility of the metabolomics data.

Unsupervised PCA was employed to gain an overview and assess quality of the GC–MS data of all the samples and interpret the data set resulting from metabolite profiles of control and MYXV infected cancer cells. For all three cell lines, PCA showed excellent separation within small in-group variability suggesting strong group effects (Fig. 2), i.e., readily discernable changes in metabolism in all cancer cell lines. Supervised PLS‐DA was employed to construct the pattern recognition and classification models of samples between control and MYXV infected groups. PLS-DA scores plots of H446, A549 and SFxL (Fig. 3) clearly showed that the profile of control and MYXV infected cells are separated from each other based on a relatively limited selection of metabolites. The clustering among samples within a group was excellent, which led to accurate descriptors of group membership based on hierarchical clustering (Fig. 4). The cross-validation approach determined the fitting of model and number of components required to build the PLS-DA model. Positive R^2^ and Q^2^ indicate that the PLS-DA model is not overfitted whereas the high R^2^ and Q^2^ (> 0.60) and the closeness between both parameters in PLS-DA model indicate that the components 1 and 2 are sufficient to construct a statistical model that correctly classifies MYXV infected cells versus controls. The most important metabolites on the VIP plot in Fig. 3B, showed the levels of both fatty acids and TCA cycle intermediates were higher in H446 control cells, while in SFxL cells (Fig. 3F), only fatty acids levels were elevated in control cells. In contrast, levels of metabolites associated with energy metabolism were elevated in MYXV infected A549 cells (Fig. 3D). Taken together, it is clear that MYXV infection causes strong but disparate changes in pathways associated with energy metabolism at the measured infectivities. The significantly different metabolites between control and MYXV infected H446, A549, and SFxL cells (Figures S1–S3) were used to construct the metabolic network of the most significant pathways through enrichment analysis for individual cancer cell line.

All cancers exhibit altered energy metabolism^28^. As such, it is unsurprising that the Warburg effect, an adaptation that supports tumor proliferation^29^, is a prominent entry in MSEA for all three cancer cell lines. Small cell lung cancers have been shown to exhibit changes in proline (and associated glutamate) metabolism. Specifically, it has been shown in H446 cells that proline starvation hinders tumorigenesis^30^ .Interestingly, in MYXV infected H446 cells, proline levels are higher in the infected cells with concomitant increase in glutamate levels (Figs. 4, S1). Similarly, non-small cell lung cancers have been shown to have an adaptation leading to altered glutathione metabolism^31^. These previous findings are confirmed by MSEA of the significantly different metabolites between A549 control and MYXV infected cells, which showed that glutathione metabolism had the greatest enrichment ratio (Fig. 5B) and that significantly lower levels of glycine, glutamate, cysteine, and pyroglutamate in infected cells were driving the detection of glutathione metabolism (Figure S2). In SCLC H446 cells, citric acid cycle (CAC) metabolites are higher in control versus treatment, whereas in control NSCLC A549 cells, they are depleted versus treatment (Fig. 4). Without separate experiments using isotope tracers, up or downregulation of the CAC cannot be readily determined. However, energy metabolism is obviously perturbed by myxoma infection.

Glioblastoma exhibits altered phospholipid metabolism^32^ depending on the specifics of microenvironmental conditions. It has been demonstrated that increased phosphatidylethanolamine-binding protein expression is a reliable marker of glioma grade^33^. Further, lipid accumulation and oxidation has been demonstrated to aid in tumor proliferation^34^. A comparison between control and MYXV – infected groups of SFxL cells show substantial differences in metabolites involved in fatty acid metabolism as evidenced by the significantly lower levels of oleic acid and vaccenic acid in infected SFxL cells (Fig. 3 and S3). Further, glioblastoma cells are known to maintain elevated levels of cholesterol^35^, an aspect that has been suggested as potential therapeutic target. Infection with myxoma virus causes a drop in cholesterol levels in SFxL levels (Fig. 4) suggesting a possible mode of oncolytic action that could be synergized with interventions targeting cholesterol metabolism.

Although there are major differences between different types of cancers, one of the distinguishing features of cancer cells is the metabolic alterations exhibited in comparison with healthy cells^36^. Breast cancer biomass has been shown to be supported by ammonia recycling via glutamate metabolism^37^. Similarly, several cancers have been shown to efficiently utilize glutamine as a metabolic adaptation along with the prevalence of Warburg metabolism^29^. Analysis of metabolites that significantly vary between control and myxoma infected cells across all three cell lines show substantial changes in the levels of lactate, amino acids (asparagine, glutamine, glycine, and serine) and oleic acid. In congruence with the changes in metabolite levels, MSEA shows that infection by myxoma has maximal effect of pathways covering ammonia recycling, glutamate/glutamine metabolism and glutathione metabolism (Figs. 5, S4–S6) in all three cell lines.

The role of biomarkers is significant in cancer detection and treatment monitoring^38^. As such, an easy to detect biomarker reporting on oncolytic changes due to MYXV infection has the potential to inform on the progression of oncolytic viral therapies. Ideally, such a biomarker will also be same or similar across different types of cancers. In the current study, six metabolites were significantly different across all three cell lines (Fig. 6), of which, four metabolites (lactate, oleic acid, asparagine, and serine) showed the same response to myxoma infection. The relevance of these metabolites stands to show specific virus infection effects since these metabolites were found to be significantly different from the respective control cell lines. Additionally, a cell-line dependent decrease in fatty acids, such as myristic, palmitic, stearic acid and vaccenic acid (Fig. 3, S1–S3), suggesting a disruption in fatty acid metabolism due to MYXV-infection was also evident. Again, without tracer experiments, it is impossible to determine if these decreases are related to uptake or utilization. These six potential biomarkers show modulation across diverse pathways such as central energy metabolism, one-carbon, and asparagine metabolism. Further, since lactate, oleic acid, asparagine, and serine are stable under normal conditions and easily detected in GC–MS (and other analytical techniques), these metabolites are candidates as reliable markers of MYXV inducted changes to cancer metabolism. Lactate stands out as a biomarker that can also be imaged using magnetic resonance^39^. Further research will need to address the specificity of these biomarkers (e.g. viral and cancer specificity), and, in general, confirm the hypothesis that these metabolites could specifically herald oncolytic viral activity.

The six metabolites found to be significantly different between the control and infected cells of all three cell lines may play an important role in mitigating tumor cell death induced by MYXV infection. Asparagine has been shown to suppress apoptosis and mediate the adaptation to glutamine depletion in cancer cells^40^. Glutamine has served a primary role in metabolic adaptation in cancer by serving as an essential energy and nutrient source, especially for glutamine dependent A549 and SFxL cells which have respectively been shown to have a ~ 33% and ~ 50% growth reduction when cultured in glutamine deficient media (the effects of glutamine deprivation in H446 are not clear)^41–43^. Serine has been shown to support cancer cell survival by mediating amino acid transport, combating oxidative stress, and promoting nucleotide synthesis. Additionally, serine uptake and synthesis has been observed to be upregulated in cancer cells^44^. Similar to serine, upregulation of glycine synthesis can potentiate and drive tumorigenesis through one carbon metabolism^45^. Oleic acid has been shown to promote cell proliferation^46^. Lactate production and excretion is a signature of cancer metabolism and serves an important role in the acidification of the microtumor environment which promotes invasion and metastasis^47^. In the context of myxoma infection, the viral machinery may disrupt central metabolic processes to an extent that significantly reduces lactate production. While it is difficult to dissect the relevance of these metabolites to cancer metabolism or myxoma virus infection, we believe that the already dysregulated metabolic processes in cancer may be disrupted by myxoma infection to allow for tumor cell death. All six of the common metabolites identified in this study are involved in potentiating cancer cell survival, so it makes sense that some may be upregulated to combat the negative effects of infection, while others may already be downregulated by infection preceding eventual cell death.

**Figure 8:**
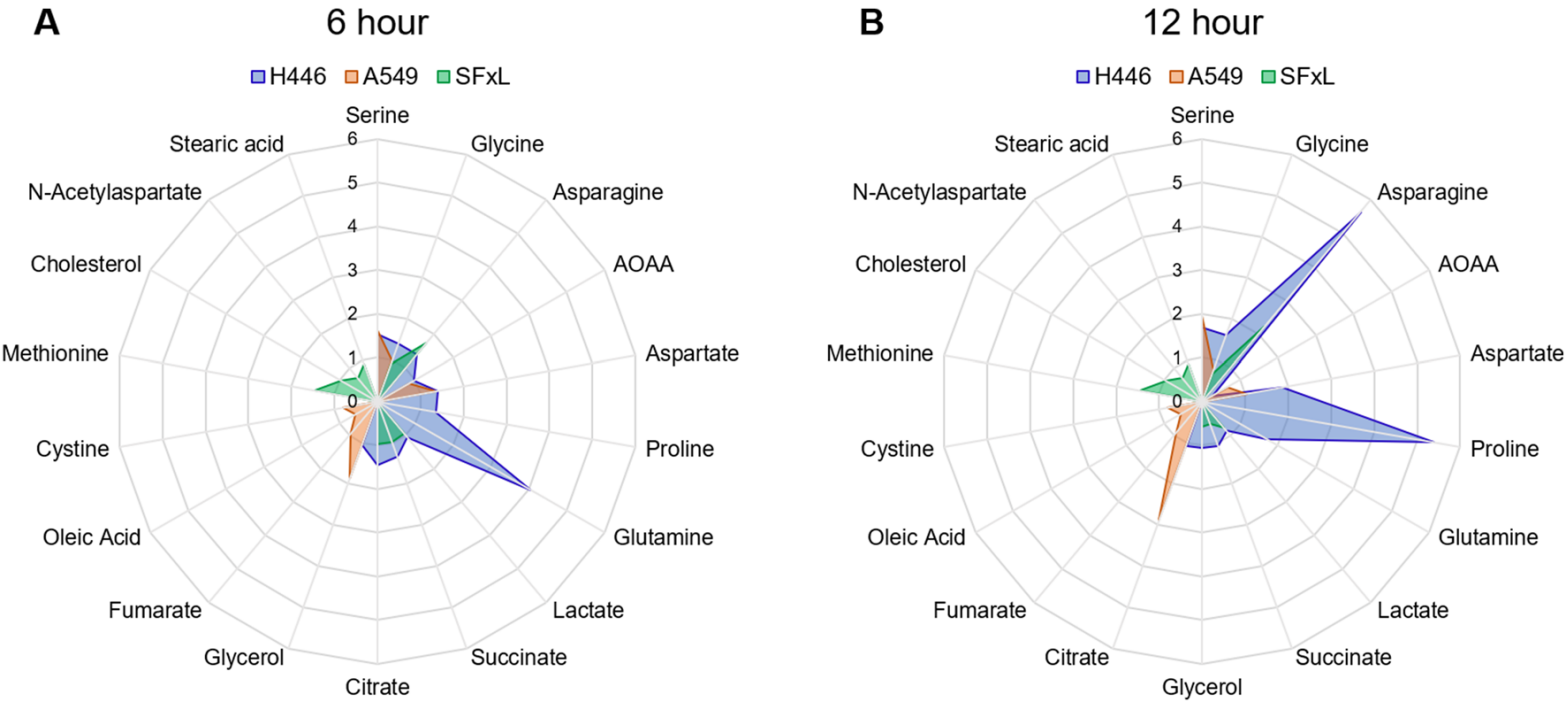
Metabolic profiles between 6 and 12 h post infection: Radar plots demonstrating the extent of changes observed in metabolite levels across 6 and 12 h of infection. The metabolites presented were selected based on the top 25 VIP scores and conservation across time points. The scale of the plots were kept the same to highlight the range of changes between the two time points for all three cells (H446, A549, SFxL). Note that H446 cells had the highest measured infectivity, as well as the greatest change in the metabolic profile versus control cells at both time points.

Another significant variable is the change in metabolism as a function of the level of infectivity. To address the infectivity dependent changes in metabolite levels through the course of infection, we plotted changes in the levels of metabolites ranked in the top 25 by VIP scores and conserved between 6 and 12 h post infection (Fig. 8). The metabolite levels were normalized to control so that the effects of infection would be highlighted across time. Comparing the differences in the metabolite profiles of each cell line at 6 and 12 h showed that in infected H446 cells glutamine levels drastically decreased while asparagine and proline increased from 6 to 12 h, indicating that greater susceptibility to infection marks greater changes in the metabolite profile. Both A549 and SFxL cells showed more subtle changes in their metabolite profiles from 6 to 12 h and interestingly both cell lines shared similar trends for glycine, one of the biomarkers identified (Fig. 6). An overall comparison of the full metabolite profiles of 6 and 12 h post infection cells showed that more fatty acids were detected at 12 h. As fatty acids were one of our identified biomarkers, we hypothesize that MYXV infection leads to a greater dysregulation of fatty acid metabolism over time. However, we note that our FBS was not dialyzed which could contribute to such changes. We plan on using dialyzed FBS for future studies.

When comparing our data to previous studies with oncolytic adenovirus infection, we observe a different effect in glucose uptake and lactate production^48^. This difference is likely due to differences in the mechanisms of myxoma and oncolytic adenovirus infections, an important control for viral metabolic effects. Our study shows that glucose uptake and lactate secretion is reduced in myxoma virus infected cells, whereas Dyer et al. show that glucose uptake and lactate secretion is increased^48^. Additionally, it is difficult to directly compare myxoma infection with oncolytic adenovirus given that they have different effects on cell viability over time. In our study there was a drastic decrease in cell viability after 24 h. Moreover, previous work on oncolytic viruses and fatty acid metabolism shows that β-oxidation of palmitate is required to fuel the TCA cycle and overall energy production for viral infection^49^. While our focus was not strictly on β-oxidation, our data suggests some common findings such as an elevation in fatty acids in control H446 and SFxL cells, indicating that β-oxidation may be upregulated in the respective infected cells. We also observed that TCA cycle intermediates were elevated in control H446 cells, which is consistent with previous reports of changes in TCA cycle metabolism on infection^49^. Additionally, we found that metabolites associated with energy metabolism were elevated in A549 infected cells, which may point to increased energy demands during viral infection^50^. Similar to Teferi et al.^50^ our data shows that glucose consumption in myxoma infected cells is not significantly different than control cells.

Overall, we have shown that several dramatic changes occur in cancer metabolism at an early stage of oncolytic myxoma virus infection. Although carrying out this work in cell culture has enabled us to characterize changes in metabolism that are specific to cancer cells, this aspect is also the primary limitation of this study. Future work will need to address more complex aspects of cancer such as utilizing hypoxic environment and tumors implanted in animal models along with biochemical characterization of signaling pathways. Ideally, future experiments will be executed using matched levels of infectivity instead of a standardized time point, but a real time method for estimating infectivity was not available to our lab at the beginning of our experimental paradigm.

## Conclusions

In conclusion, we have broadly identified global changes in metabolism caused by myxoma infection in three different cancer cell lines. In order to obtain mechanistic insights into MYXV targeting of cancer cells, further studies using stable isotope tracers will need to be performed. Since cancer cells are able to utilize a wide range of substrates, they are amenable to study using various ^13^C^51^ or ^2^H stable isotope^52,53^ tracers. These studies could also be carried out with the addition metabolic modulators or other existing therapeutic agents. Experiments utilizing ^13^C enriched substrates utilizing a combination of NMR spectroscopy and GC–MS are already underway.

## Materials and methods

### Cell lines, chemicals, and media

Lung carcinoma (A549) cell line was procured from American Type Culture Collection (Manassas, VA, US). H446 (small cell lung cancer) was provided by Dr. Maria Zajac-Kaye, University of Florida. Glioblastoma SFxL cell line was received as a gift from Dr. Ralph Deberardinis, University of Texas Southwestern Medical Center. Dulbecco’s Modified Eagle Medium was purchased from Millipore Sigma (St. Louis, MO, US), Phosphate Buffered Saline and trypan blue were acquired from Fisher Scientific (MA, US) and Fetal Bovine Serum was bought from Atlas Biological (Fort Collins, CO, US). Methoxamine hydrochloride (MOX) and N-methyl-N-(tert-butyldimethylsilyl)trifluoroacetamide (MTBSTFA) with tertbutyldimethylchlorosilane (TBDMS) for derivatization were procured from Thermo Fisher Scientific, (Waltham, MA, US).

### Cell culture and myxoma virus infection

H446, A549, and SFxL cell lines were maintained in a growth medium containing Dulbecco’s Modified Eagle Medium, 10% v/v FBS, penicillin and streptomycin (50 µg/mL each), and 10 µg/mL neomycin. Cell lines were incubated at 37 °C in air (95%) and CO_2_ (5%) atmosphere in an incubator (Heracell Vios 160i). Growth media was replenished every 3 days and once cells reached at 80% confluence, cells were subcultured (1:10) into six 100 mm OD (culture area = 56.7 cm^2^) culture plates. For viral infection, the cell monolayers grown to ~ 70% confluency. Cells were washed with warm PBS and incubated with DMEM (control group) and wildtype myxoma virus in DMEM (infected group) for 2 h. The stocks of myxoma virus were generated using published procedures^54^ and a multiplicity of infection (MOI) of 5:1 was utilized. The MOI was based on the viral titer of MYXV infected rabbit kidney epithelial cells (RK-13)^55^, which were used to propagate the MYXV stocks. In order to minimize the possibility of changes in metabolism due to cell death and virus replication as opposed to viral mediated changes in metabolism^56,57^, 6 and 12 h infection time points were chosen. After 2 h of the Myxoma virus infection, cells were washed once with warm media to remove excess virus and incubated for 6 and 12 h. After the incubation, media was collected, and cells were trypsinized, resuspended in DMEM with FBS (10 mL total volume) and counted on a standard hemocytometer using trypan blue. Cells were pelleted by centrifugation (350×*g*) at 4 °C. Residual media was removed by washing three times with ice-cold PBS and subsequent centrifugation each time. Cell pellets were stored at − 80 °C until analysis.

### GC–MS sample preparation

Cell pellets were extracted with 1 mL of Acetonitrile:Isopropanol:Water (3:3:2, v:v:v), and centrifuged for 15 min at 10,000×*g*. The supernatant containing metabolites was transferred into a new tube and dried down completely. Dried cell extract was dissolved in 0.5 mL of Acetonitrile:Water (1:1, v:v) and centrifuged for 5 min at 10,000×*g*. The supernatant from the second step was transferred into GC reaction vial (Reacti-vial) and dried down under N_2_ gas air flow. Media samples were directly loaded (10 μL) to GC reaction vials and dried. Fifty microliters of MOX was added to dried cell extracts and dried media in GC vials and incubated at 30 °C for 1.5 h on a heating block. MTBSTFA + TBDMS reagent (50 µL) was added to each vial and again incubated for 1 h at 60 °C. Finally, reaction vials were centrifuged and the supernatant was transferred into the insert for GC–MS analysis.

### Gas chromatography–Mass spectrometry (GC–MS) analysis

GC–MS data was acquired with Single Quadrupole Mass Spectrometer and Gas Chromatograph (Trace 1310) (Thermo Fisher Scientific, USA). The GC column was 30 m long dimethyl (95%)/diphenyl polysiloxane (5%) RTX-5MS with 0.25 mm ID, film of 0.25 µm, and guard column (10 m) (Restek, PA, USA). Starting temperature of GC oven was 60 °C for 60 s and the temperature increased to 325 °C (@10 °C/min) and final hold time was 5 min. The MS ion source temperature was 230 °C. Helium acts as carrier gas.

### Peak integration and metabolite identification

Metabolites in cell samples were identified using NIST mass spectral library with Xcalibur software (version 4.1). Total ion current (TIC) chromatogram based peak areas of identified metabolites were extracted for each of the samples and combined in a tabular form for statistical analysis. Peak areas were integrated using Xcalibur software library batch processing method with ICIS peak fitting, height cutoff at 5.0% of the peak, and a S/N cutoff threshold of 3.0.

### Statistical analysis

The peak intensity of metabolites was imported to online MetaboAnalyst 5.0 and normalized by the sum of intensities followed by generalized log10 transformation and pareto scaling to provide equivalent weight among the variables. The web-based MetaboAnalyst 5.0^58^ was employed to perform t-tests, unsupervised principal component analysis (PCA), partial least square discriminant analysis (PLS-DA) (a supervised method), and Hierarchical Clustering analysis^58^. The Q^2^ and R^2^ values. were calculated to assess the robustness of PLS-DA model. The Variable Importance in Projection (VIP) obtained from PLS-DA was utilized to extract the importance of various metabolite to the model. Compounds with a VIP scores > 1 is considered to be influential in PLS-DA. The significantly different metabolites between control and infected cells were plotted using in-house python scripts and similar metabolites were used for Metabolite Set Enrichment Analysis (MESA). *P* ≤ 0.05 was considered significant for individual metabolites as well as MSEA.

### Percent infectivity analysis

Myxoma virus infectivity was assessed over a 24 h period. Cells were handled exactly as in the cell culture and Myxoma virus infection section. Cells were imaged on a Revolve hybrid microscope obtained from ECHO (CA, US). Images were captured at 0 h, 6 h, 12 h, and 24 h using a FITC and transfluorescent module. Cell counts were performed on ImageJ (NIH, MD, US) to determine the number of GFP positive cells and the number of total cells present in each respective image. Cell numbers were used to calculate percent infectivity at each time point.

### Glucose consumption measurement

An Evencare G2 glucose meter was utilized to measure the glucose level in media samples. Amounts of residual glucose in control and infected cell media samples were determined by subtracting the raw glucose measurement from time zero samples.

## Supplementary Information

Supplementary Information.

## Data Availability

Data generated or analyzed in this study are included in this manuscript and its Supporting Information.
