# Supplementary material for: Metabolic signatures associated with oncolytic myxoma viral infections
Source: Sci Rep. 2022 Jul 23;12:12599. doi: 10.1038/s41598-022-15562-3 (PMC9308783; doi:10.1038/s41598-022-15562-3)
Supplement: Supplementary file 1 — Supplementary Information. [file 41598_2022_15562_MOESM1_ESM.pdf]

Metabolic Signatures Associated with Oncolytic Myxoma Viral Infections

Rohit Mahar[1,†], Mukundan Ragavan[1,†], Mario C. Chang[1,†], Savannah Hardiman[2], Nissin Moussatche[2], Adam Behar[1], Rolf Renne[2], and Matthew E. Merritt[1,*]

[1]Department of Biochemistry and Molecular Biology, University of Florida College of Medicine, Gainesville, Florida, U.S.A.; rmahar@ufl.edu (RM), mukundan@ufl.edu (MR), marioc14@ufl.edu (MCC), beharadam5@gmail.com (AB), matthewmerrit@ufl.edu (MEM).
[2]Department of Molecular Genetics and Microbiology, University of Florida College of Medicine, Gainesville, Florida, U.S.A.; shardiman@ufl.edu (SH), nissin@ufl.edu (NM), rrenne@ufl.edu (RR)

[†] these authors contributed equally
*Correspondence: matthewmerritt@ufl.edu

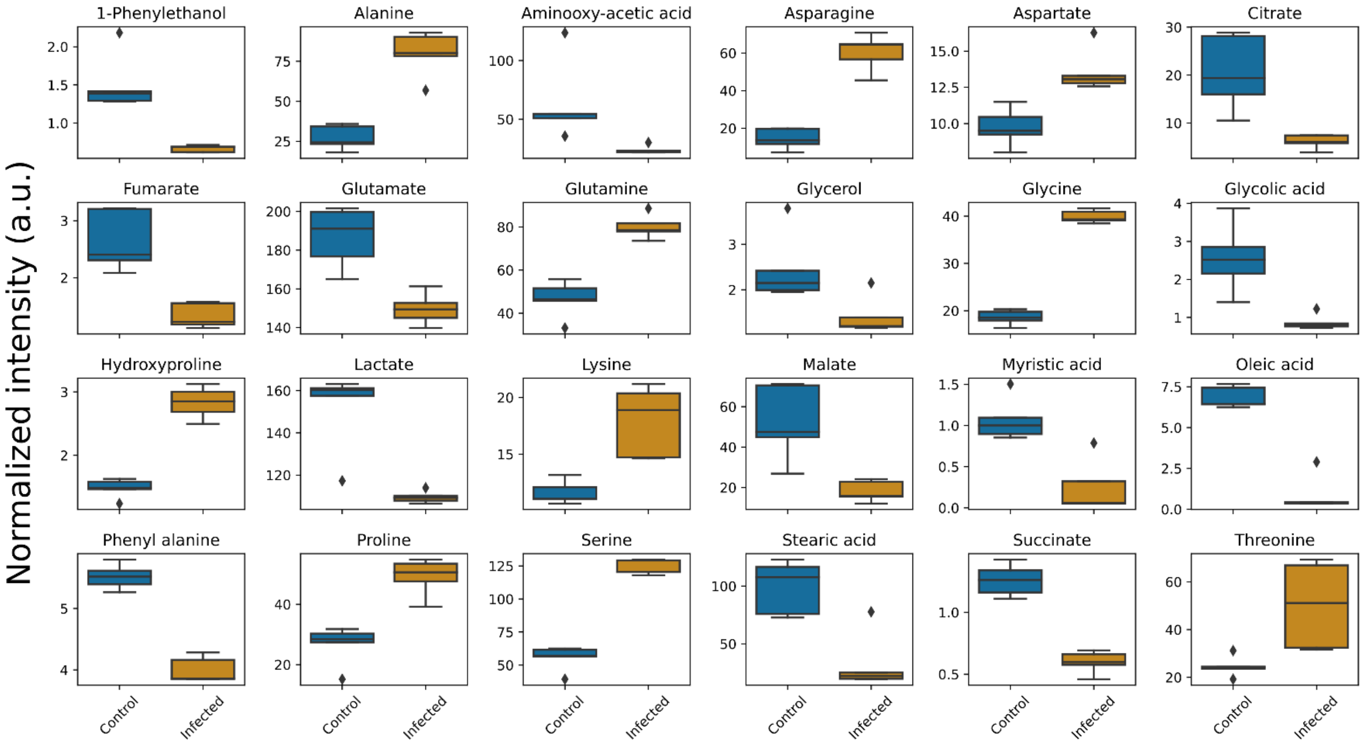

Supplementary Figure S1: Differential metabolite levels between control and infected H446 cells: Box and Whisker plots displayed the differential level of significantly different metabolites between control and infected H446 cells. Significance levels of metabolites between control and treated groups were determined using GC-MS data, employing statistical analysis via t-test in MetaboAnalyst. $P \leq 0.05$ was considered statistically significant between control and infected H446 cells (N=5). GC-MS intensities shown here were normalized to sum.

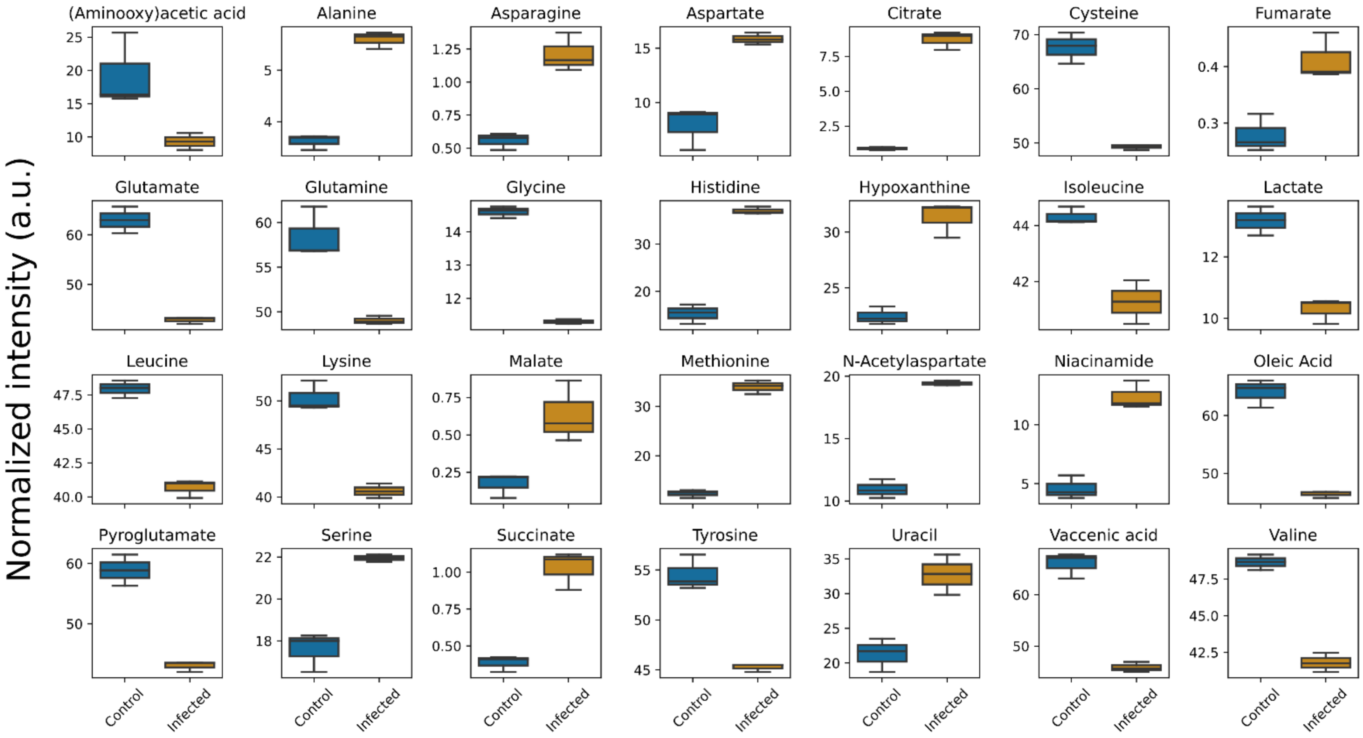

Supplementary Figure S2: Differential metabolite levels between control and infected A549 cells: Box and Whisker plots displayed the differential level of significantly different metabolites between control and infected A549 cells. Significance levels of metabolites between control and treated groups were determined using GC-MS data, employing statistical analysis via t-test in MetaboAnalyst. P≤0.05 was considered statistically significant between control and infected A549 cells (N=3). GC-MS intensities shown here were normalized to sum.

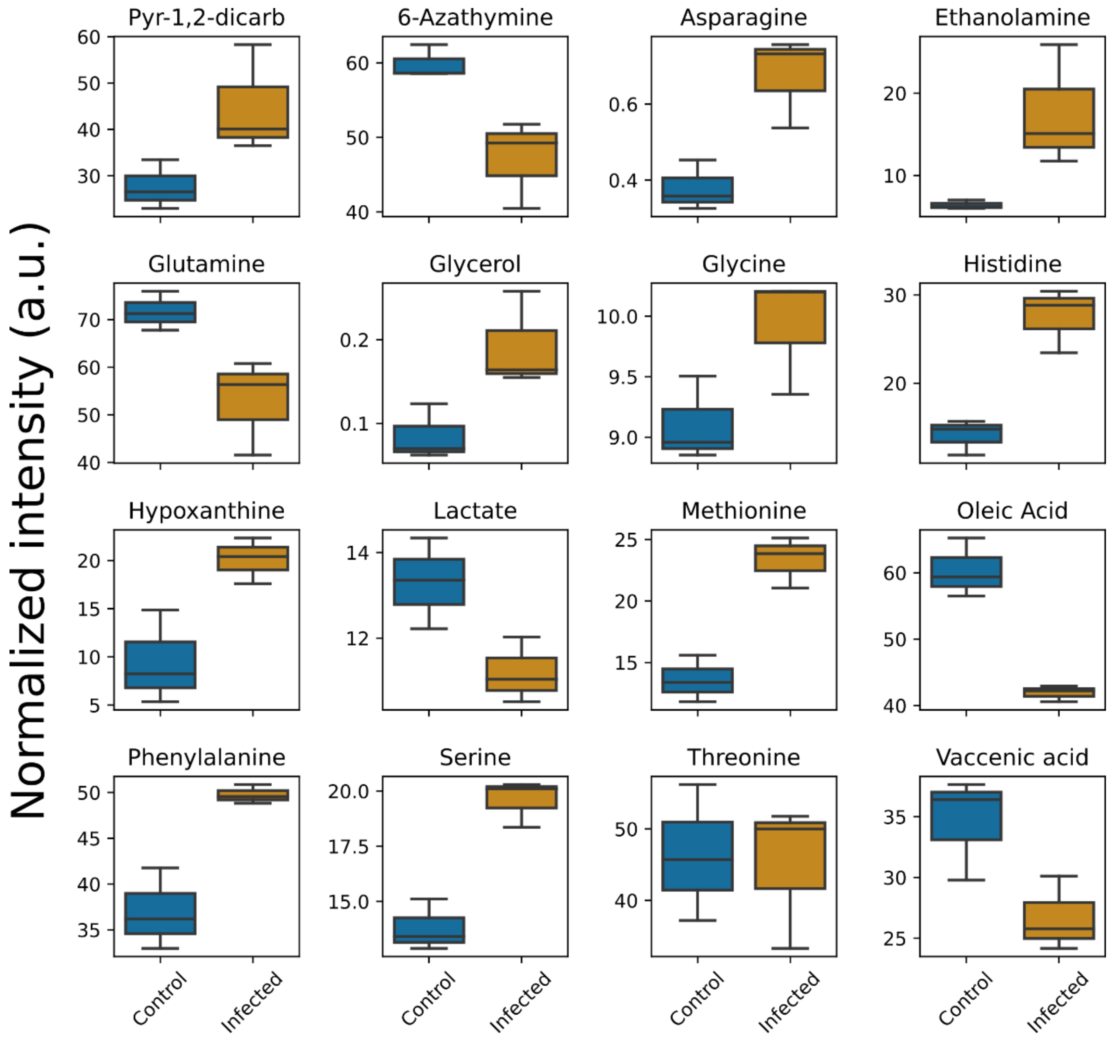

Supplementary Figure S3: Differential metabolite levels between control and infected SFxL cells: Box and Whisker plots displayed the differential level of significantly different metabolites between control and infected SFxL cells. Significance levels of metabolites between control and treated groups were determined using GC-MS data, employing statistical analysis via t-test in MetaboAnalyst. $P \leq 0.05$ was considered statistically significant between control and infected SFxL cells (N=3). GC-MS intensities shown here were normalized to sum.

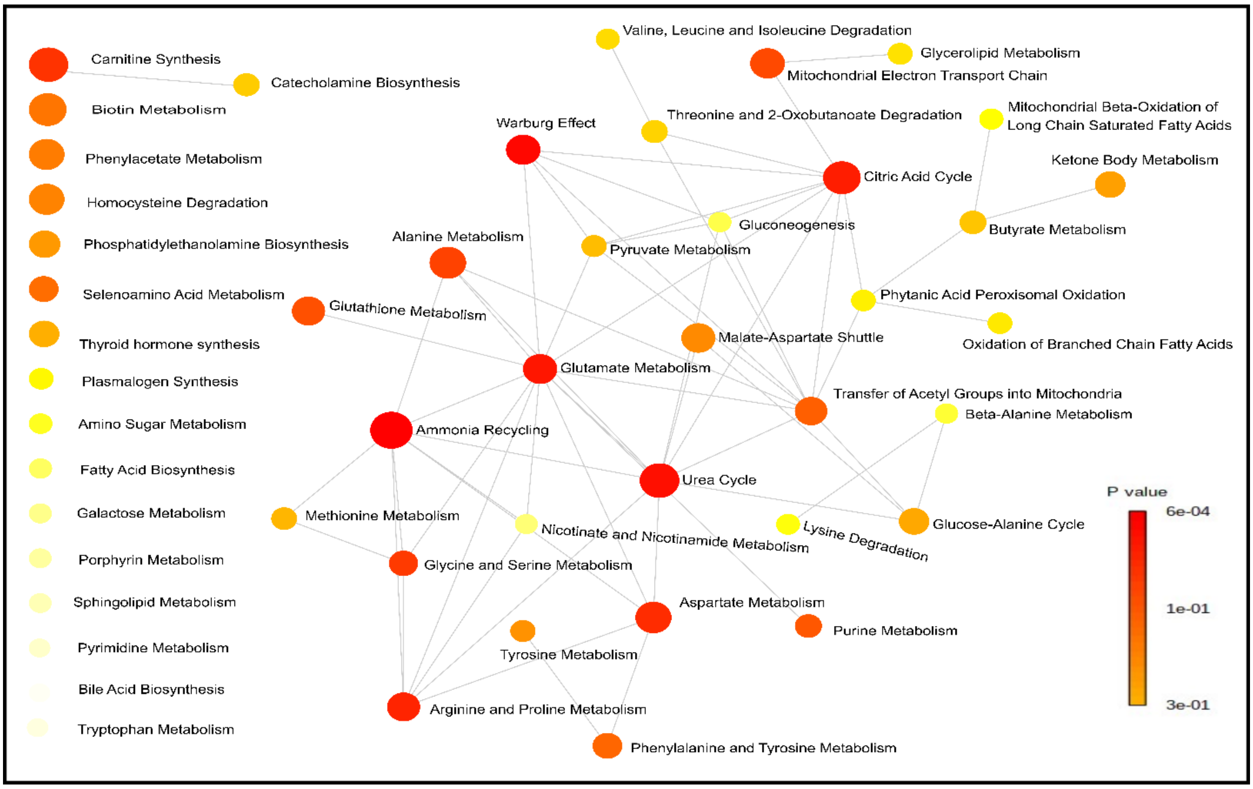

Supplementary Figure S4: Metabolite Set Enrichment Analysis of H446 cells: Interactive network of pathways showing the connections between pathways and individual pathway. Significantly different Metabolites between control and infected H446 cells were used for metabolite set enrichment analysis.

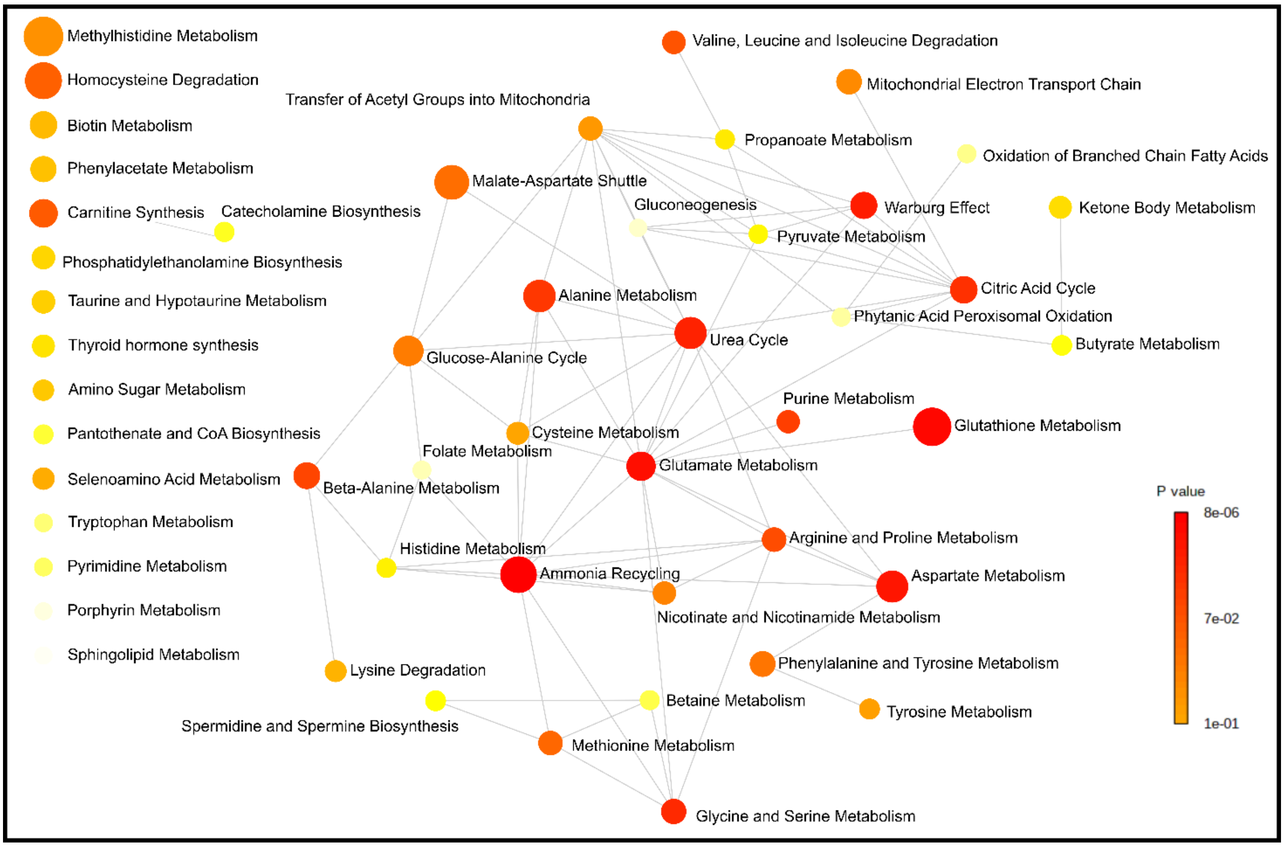

Supplementary Figure S5: Metabolite Set Enrichment Analysis of A549 cells: Interactive network of pathways showing the connections between pathways and individual pathway. Significantly different Metabolites between control and infected A549 cells were used for metabolite set enrichment analysis.

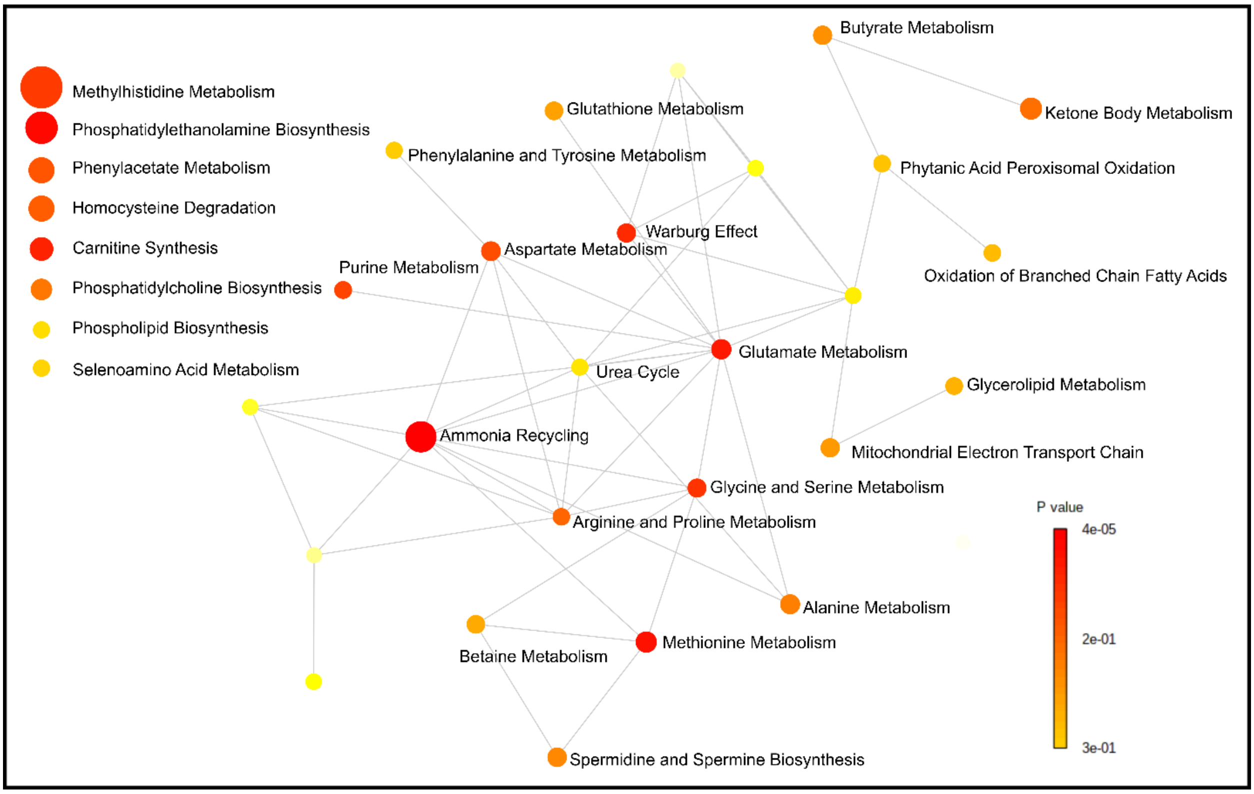

Supplementary Figure S6: Metabolite Set Enrichment Analysis of SFxL cells: Interactive network of pathways showing the connections between pathways and individual pathway. Significantly different metabolites between control and infected SFxL cells were used for metabolite set enrichment analysis.

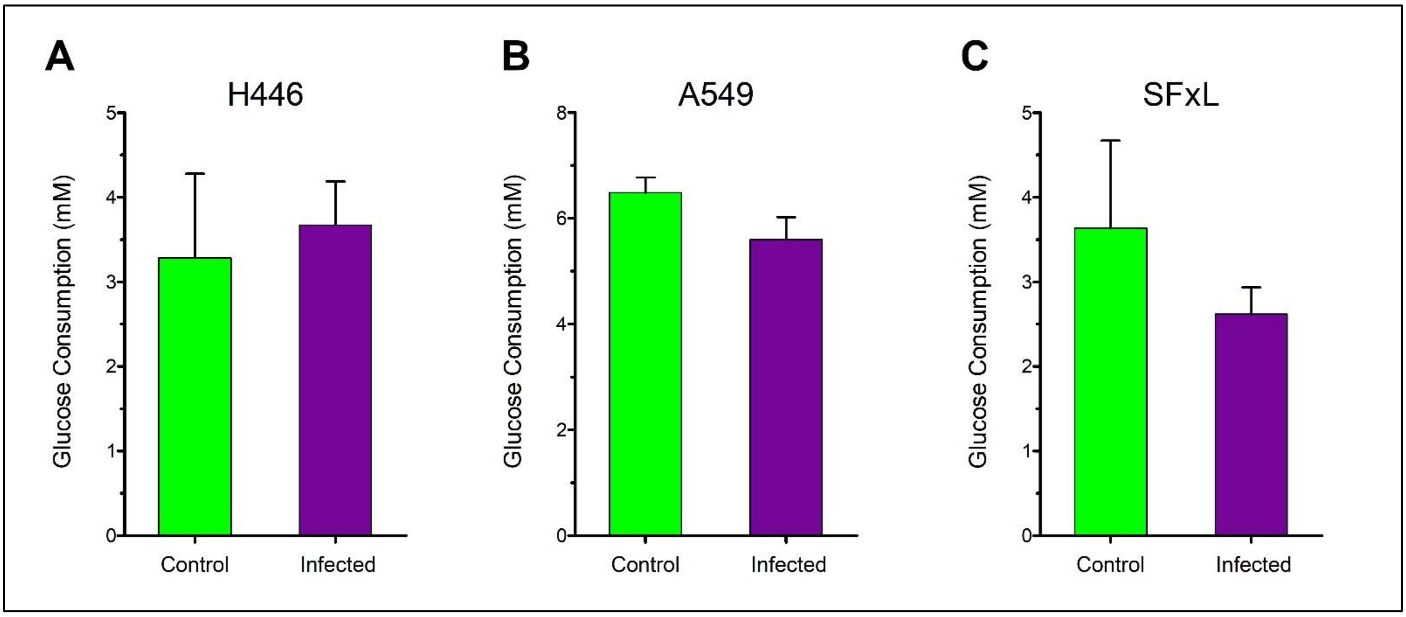

Supplementary Figure S7: Control and infected cell glucose consumption: Glucose consumption was assessed at the 12 hour time point for all three cell lines. H446 (A) showed no apperent difference between control and infected cells, while A549 (B) and SFxL (C) showed lower glucose consumption in the infected cells. (Data is presented as mean ± SEM for biological triplicates. Statistical analysis was performed with Student's t-test and no significant difference was observed).

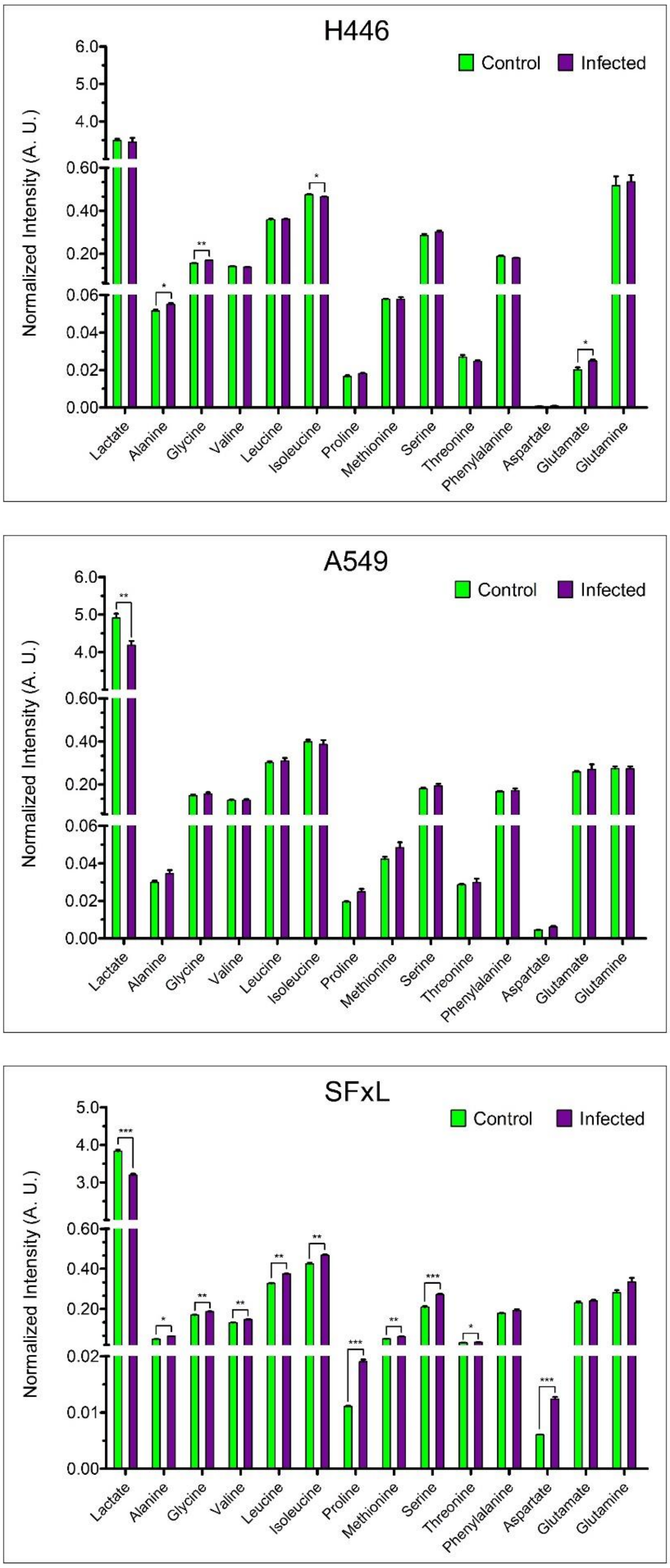

Supplementary Figure S8: Panel of extracellular metabolites show differences between control and infected cells: GC-MS metabolic panel of media samples show differences in extracellular metabolites between control and infected H446 (top), A549 (middle), and SFxL (bottom) cells, at 12 hour time point of the infection. (Data is presented as mean ± SEM for biological triplicates. Statistical analysis was performed with Student's t-test and is presented as: '*' if P≤0.05, '**' if P≤0.01, '***' if P≤0.001).

Supplementary Table S1: List of all metabolites detected by GC-MS across three cell lines (H446, SFxL, and A549)

Metabolite	m/z Quantitation ion
Alanine	260
Asparagine	417
Aspartate	418
Cholesterol	443
Citrate	591
Cysteine	448
Cystine	348
Ethanolamine	232
Fumarate	287
Glutamate	432
Glutamine	431
Glycerol	377
Glycine	246
Glycolic acid	247
Histidine	440
Hydroxyproline	416
Hypoxanthine	307
Isoleucine	302
Lactate	261
Leucine	200
Lysine	431
Malate	419

Methionine	320
Myristic acid	285
N-Acetylaspartate	460
Niacinamide	122
Oleic acid	339
Palmitic Acid	313
Phenylalanine	378
Proline	258
Pyroglutamate	300
Pyruvate	139
Serine	390
Stearic acid	303
Succinate	289
Threonine	417
Tyrosine	508
Uracil	325
Vaccenic acid	339
Valine	302
(2R)-Pyrrolidine-1,2-dicarbox-ylate	330
(Aminooxy)acetic acid	262
1-Phenylethanol	122
4,6-Dihydroxypyrimidine	283
6-Azathymine	298
